# NIEHS Responds to Katrina

**DOI:** 10.1289/ehp.114-a28

**Published:** 2006-01

**Authors:** Renée Twombly

NIEHS director David Schwartz knows firsthand what the country’s worst natural disaster looks like. Within days of Hurricane Katrina’s winds and waves, he led an advance medical team of 50 physicians, nurses, and health care workers from the NIH, the NIEHS, and Duke University Medical Center to Mississippi to respond to the disaster. There he found “nothing short of what one would expect in a war zone,” as he wrote on the NIEHS website when he returned two weeks later. The extent of destruction was “overwhelming, with cars upturned, tractor trailers scattered like matchsticks, homes completely leveled, buildings destroyed.”

Schwartz was just one of many NIEHS specialists who were, and in some cases still are, part of the largest disaster response mobilization in U.S. history. The institute’s response to Katrina involved quick, extensive planning and organization within the NIEHS and across a span of sister agencies, such as the NIH, the Environmental Protection Agency (EPA), the Occupational Safety and Health Administration (OSHA), the Centers for Disease Control and Prevention (CDC), the Department of Defense, the Food and Drug Administration, the U.S. Department of Agriculture (USDA), and the Department of Homeland Security and Federal Emergency Management Agency (FEMA).

“Katrina was an environmental health catastrophe, and [Hurricane Rita a month later] just added to the damage,” says Allen Dearry, the NIEHS associate director for research coordination, planning, and translation, who has acted as the institute’s response coordinator. “The institute’s expertise is connecting environmental exposure to human health, and there are bigger questions as the result of this natural disaster than we have encountered before.”

## Immediate Response on Many Fronts

The NIEHS went into action shortly after Katrina hit. On August 31, the day after the New Orleans levees broke, Joseph “Chip” Hughes and the team he directs at the NIEHS Worker Education and Training Program (WETP) developed a PowerPoint safety awareness training primer for first responders and posted it on the NIEHS website. The group had produced 11 versions of the primer by October 27, updated as the scope of the disaster unfolded to include information on such health threats as trench foot, waterborne diseases, and mold. The primer—available in English, Spanish, and Vietnamese (since there are many Vietnamese in the Gulf Coast region)—has been downloaded at least 1,600 times, and more than 35,000 printed copies have been distributed. The WETP team has also delivered hands-on hazards training to federal employees and federally employed contractors in the field in Mississippi, Louisiana, Alabama, and Texas.

Just as human health was at risk, so was that of the animals left stranded by the hurricane. Starting September 7, William Stokes, director of the National Toxicology Program (NTP) Interagency Center for the Evaluation of Alternative Toxicological Methods, who also serves as the chief veterinary officer of the U.S. Public Health Service, headed the federal effort to assist with the rescue and shelter of those animals. Stokes led an initial team of 10 veterinarians and a public health nurse whose number quickly doubled to meet the overwhelming needs of two emergency animal shelters, one located on the Louisiana State University Baton Rouge campus, and one at a livestock exposition center in Gonzales. The shelters’ residents included carriage horses from New Orleans, a pet alligator, an eight-foot-long python, pot-bellied pigs, birds, turtles, and a variety of other pets. A total of 35 Public Health Service veterinarians and countless volunteers examined and treated more than 5,000 creatures, inserted identifying m i c r o c h i p s , t o o k photographs, and moved many of the animals out to other shelters to await their owners.

“In addition to keeping all of these animals healthy, our goal was to ensure that as many as possible were returned to their owners in order to avoid further stress from the pet loss on top of all their other losses,” says Stokes. He adds that in the future, he hopes evacuation policies will allow for animals to accompany their owners.

## Meeting of the Minds

As the extent of the disaster unfolded, the NIEHS continued to send out experts to assist other federal agencies. Mary Wolfe, director of the NTP Office of Liaison and Scientific Review, was sent to CDC headquarters in Atlanta for five days in mid-September to help assimilate field data from teams along the Gulf Coast who were assessing emerging health threats. Sam Arbes, an epidemiologist in the NIEHS Laboratory of Respiratory Biology who studies the health effects of mold, went to Baton Rouge with a CDC team to prepare a document that helped local and state officials assess environmental damage and public health issues as they planned for reentry of residents and restoration. The document addressed public health issues associated with drinking water, sewage disposal, roads and transportation, toxic exposures, housing, and schools, among other things.

NIEHS-funded environmental health sciences centers also swung into action. Immediately after the hurricane, Schwartz asked the center directors to work collaboratively to define the research questions that would surround the effects of the hurricane and the recovery of the population. Five working groups within the centers program addressed issues of worker surveillance and health, water quality and microbes, water quality and chemical contamination, mold and respiratory consequences, and outreach and education for the affected populations. The groups have since provided Schwartz with a critical assessment of the research questions that could be addressed.

Some action has begun. Staff from the centers’ Community Outreach and Education Programs have banded together to create educational and outreach materials about the hazards that the populace may find in their homes [see “COEPs Contribute to Hurricane Relief,” next article]. Centers will also be conducting pre-and postdeployment blood sampling and analysis of New York City firefighters deployed to help the relief efforts in New Orleans. And key experts from the centers have been invited by groups such as the American Red Cross to consult on environmental problems in the region that arose from the storms. They have done some sampling of water, molds, and sediment in the region.

Back home, institute staff developed an NIEHS Natural Disaster Response website to disseminate information to workers and residents about conditions in the Gulf Coast [see the EHPnet article, p. A27 this issue]. Dearry acted as a liaison with call centers set up by the NIH and the CDC, providing information on human and environmental health issues to pass along to callers. The call centers initially took calls just from health care providers, state and local environmental and health agencies, clinics, and other providers, but were soon opened to calls from the public as well.

## Long-Term Study of Environmental Health Risks

Some of the NIEHS disaster response efforts are unique programs that will help identify the environmental hazards produced by Katrina as well as provide long-term insights into the link between environmental toxicants and health outcomes. For example, the NIEHS website features a geographic information system (GIS) database that is designed to help expedite cleanup efforts, but which can be continually developed and updated as a tool to track environmental health.

Led by William Suk, director of both the NIEHS Center for Risk and Integrated Sciences and the Superfund Basic Research Program, the GIS overlays maps and high-resolution aerial photography of Texas, Louisiana, and Mississippi with a wealth of demographic, hydrographic, infrastructure, and industrial/agricultural data from publicly available sources. With the assistance of NIEHS academic partners at Duke University and the University of California, San Diego, supercomputing center, the interactive maps pinpoint the location of Superfund sites (four in New Orleans alone), scores of Toxics Release Inventory–reporting sites (those that release toxic contaminants), and the hundreds of oil and gas rigs, gas stations, chemical industries, refineries, and crude petroleum and natural gas operations in the Gulf Coast region.

Information now being collected on water and air sampling in the area will be added as a way to model the movement of contaminants and identify sources of human exposure. For example, one-quarter of the areas sampled by the EPA in New Orleans by late September showed benzene levels that were more than twice the NTP intermediate safety level. And there were hundreds of reported oil and toxicant spills—including gas that may have seeped from an estimated 350,000 swamped cars—as well as drowned industrial and toxic waste dumps. Suk and his team of institute scientists and academic partners are working 14 to 20 hours a day to pull in data from federal agencies such as the EPA, the CDC, and OSHA in order to create what he calls a “national model that can track environmental health, both for the short-term use of responders and cleanup crews and long-term assessment of health consequences.” The model is available on the NIEHS Natural Disaster Response website.

Among the resources they are tapping are the Centers for Oceans and Human Health, supported jointly by the NIEHS and the National Science Foundation. The four centers have been sampling and analyzing floodwaters from New Orleans, and received $150,000 in National Science Foundation “rapid response” funding to collaboratively investigate the health of Lake Pontchartrain, into which 100 billion liters of New Orleans floodwater has been pumped. Researchers at these centers will sample and document the presence, abundance, and fate of waterborne pathogens such as *Escherichia coli* and *Vibrio vulnificus* (which produces a cholera-like infection and is already responsible for deaths in the area) as well as heavy metals and other toxicants in the pollution plume entering Lake Pontchartrain and beyond. They will also monitor the development of harmful algal blooms that could result from matter pumped into the lake. The information will then be linked to the GIS database.

Frederick Tyson, who administers the Centers for Oceans and Human Health program, says, “We have galvanized the talents we have to give us important answers to a public health crisis that is happening right now and that will impact public health in that region.” Suk adds that Katrina has offered “an experiment that no one wanted but which we now have in place to study real problems that will allow us to gain a better understanding of environmental health risks.”

## Figures and Tables

**Figure:**
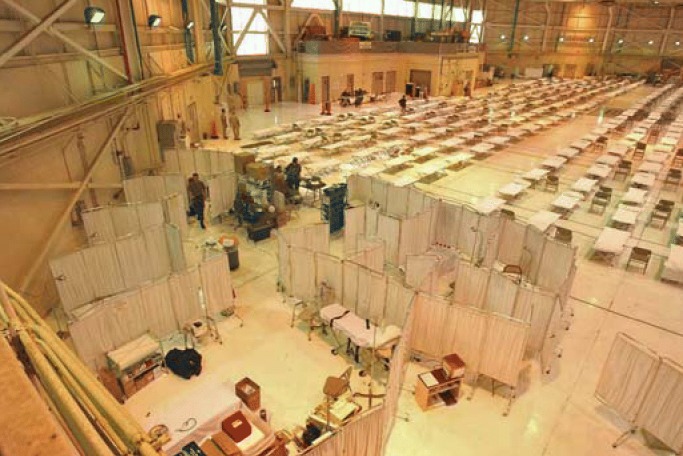
At the ready. NIEHS staff came soon after Katrina hit to help at a 500-bed field hospital in a Meridian, Mississippi, hangar.

**Figure:**
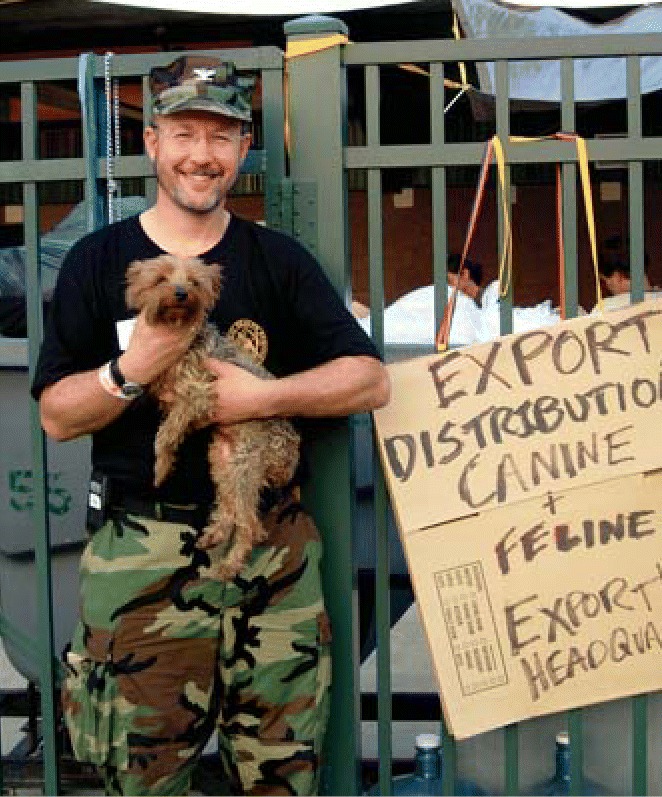
Saving man’s best friends. Bill Stokes and a team of vets and volunteers helped stranded pets.

**Figure:**
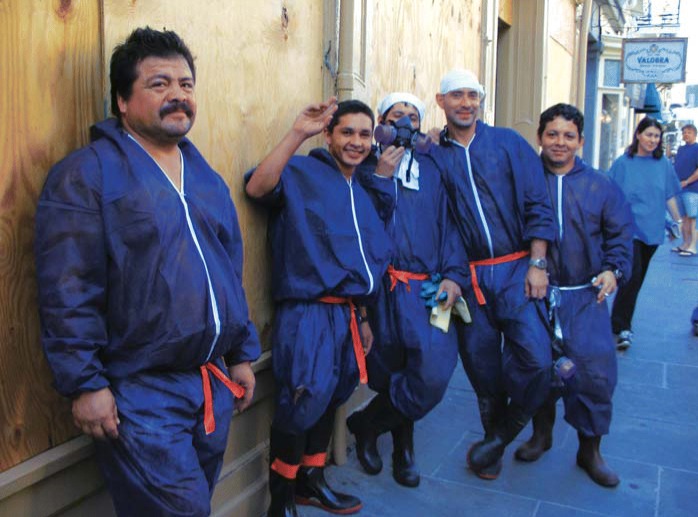
Rebuilding safely. The NIEHS WETP has developed a primer to guide construction and cleanup workers in rebuilding the Gulf Coast in a safe manner.

